# Negative Differential Friction Predicted in 2D Ferroelectric In_2_Se_3_ Commensurate Contacts

**DOI:** 10.1002/advs.202103443

**Published:** 2021-11-10

**Authors:** Jingge Sun, Lili Zhang, Rui Pang, Xing‐Ju Zhao, Jiangtao Cheng, Yimin Zhang, Xinlian Xue, Xiaoyan Ren, Wenguang Zhu, Shunfang Li, Zhenyu Zhang

**Affiliations:** ^1^ Key Laboratory of Material Physics Ministry of Education School of Physics and Microelectronics Zhengzhou University Zhengzhou 450001 China; ^2^ Key Laboratory of Strongly‐Coupled Quantum Matter Physics Chinese Academy of Sciences School of Physical Sciences University of Science and Technology of China Hefei Anhui 230026 China; ^3^ International Center for Quantum Design of Functional Materials (ICQD) Hefei National Laboratory for Physical Sciences at the Microscale, and Synergetic Innovation Center of Quantum Information and Quantum Physics University of Science and Technology of China Hefei Anhui 230026 China

**Keywords:** 2D ferroelectric materials, first‐principles calculations, In_2_Se_3_, negative differential friction

## Abstract

At the macroscopic scale, the friction force (*f*) is found to increase with the normal load (*N*), according to the classic law of Da Vinci–Amontons, namely, *f* = *µN*, with a positive definite friction coefficient (*μ*). Here, first‐principles calculations are employed to predict that, the static force *f*, measured by the corrugation in the sliding potential energy barrier, is lowered upon increasing the normal load applied on one layer of the recently discovered ferroelectric In_2_Se_3_ over another commensurate layer of In_2_Se_3_. That is, a negative differential friction coefficient *μ* can be realized, which thus simultaneously breaking the classic Da Vinci–Amontons law. Such a striking and counterintuitive observation can be rationalized by the delicate interplay of the interfacial van der Waals repulsive interactions and the electrostatic energy reduction due to the enhancement of the intralayer Se—In ionic bonding via charge redistribution under load. The present findings are expected to play an instrumental role in design of high‐performance solid lubricants and mechanical‐electronic nanodevices.

## Graphical abstract

The present study predicts that negative sliding friction coefficient can be realized in electrically polarized two‐dimensional In_2_Se_3_ commensurate contacts, which can be rationalized by the delicate interplay of the interfacial van der Waals repulsive interactions and the electrostatic energy reduction due to the enhancement of the intralayer Se‐In ionic bonding via charge redistribution under loading.

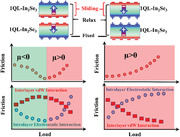

## 1 Introduction

Friction is one of the most important and significant problems in science and technologies,^[^ ^1^, ^2^ ^]^ which may play an instrumental role in diverse systems that cover from macroscopic equipment,^[^ ^3^ ^]^ through the nanometer contacts in nanomachines^[^ ^2^, ^4^ ^]^ to biological molecular motors.^[^ ^5^ ^]^ At the macroscopic scale, friction force (*f*) normally increase with increasing normal load (*N*) according to Da Vinci–Amontons law, namely, *f* = *µN*, with a positive definite friction coefficient (*µ*), and the friction force *f* is independent on other factors, such as apparent contact area, sliding velocity, etc. However, such a classic law has been demonstrated to breakdown at the microscopic scale. Research of recent decades has identified that microscopic friction becomes very complex and sensitive to various factors, such as temperature,^[^ ^6^, ^7^, ^8^, ^9^ ^]^ sliding velocity,^[^ ^3^, ^8^, ^9^, ^10^, ^11^, ^12^, ^13^ ^]^ size effects,^[^ ^14^, ^15^, ^16^, ^17^ ^]^ surface roughness.^[^ ^3^, ^18^, ^19^ ^]^ Moreover, frictions via electronic and magnetic dissipations,^[^ ^20^, ^21^, ^22^, ^23^ ^]^ radiative heat transfer,^[^ ^6^, ^24^ ^]^ and quantum effects^[^ ^25^, ^26^ ^]^ have also been respectively revealed.

In contrast to liquid lubrications, which may fail in some extreme conditions,^[^ ^27^, ^28^ ^]^ structural superlubricity has emerged as a new promising remedy for the reduction of friction,^[^ ^29^, ^30^ ^]^ which generally originates from the effective cancelation of lateral forces with incommensurate rigid crystalline contacts^[^ ^31^, ^32^ ^]^ due to the lattice mismatch and thus significantly reduces the energy barriers of motion.^[^ ^33^, ^34^, ^35^ ^]^ Such an intriguing phenomenon was originally predicted three decades ago in nanoscale homogeneous graphitic contacts^[^ ^36^ ^]^ and later termed superlubricity,^[^ ^37^ ^]^ where the friction exhibits peak and superlow feature in commensurate and incommensurate contacts, respectively. However, only recent experiments have unambiguously observed the proposed superlubricity in various 2D material incommensurate van der Waals (vdW) junctions, including homogeneous contacts of graphite nanoflake‐graphite,^[^ ^38^ ^]^ heterojunctions of graphene‐hexagonal boron nitride (hBN),^[^ ^39^ ^]^ and graphene‐MoS_2_.^[^ ^35^ ^]^ More intriguingly, adhesion‐dependent negative friction coefficients of chemically modified graphite was reported recently in experiment.^[^ ^40^, ^41^ ^]^ Note that, here the negative friction coefficient could be more rigorously expressed as negative differential friction (NDF), coined by analogy with another two very popular concepts of negative differential resistance^[^ ^42^, ^43^ ^]^ and negative differential capacitance.^[^ ^44^ ^]^ Moreover, NDF was also theoretically probed in superlubric graphene‐hBN heterojunctions,^[^ ^45^, ^46^ ^]^ graphene‐graphene,^[^ ^47^ ^]^ and ferroelectric materials.^[^ ^48^ ^]^ However, in all the previously reported systems including graphite‐graphite homo‐junction, the superlubricity and NDF were sustained either by the condition of incommensurate contacts,^[^ ^36^, ^37^ ^]^ which may be blocked to commensurate configurations during the sliding,^[^ ^37^ ^]^ or by the reduction of the potential corrugation by the vdW interactions in the attractive regime of the interfacial separation above its equilibrium.^[^ ^46^, ^48^ ^]^ Very recently, nonmonotonic interfacial friction under load is predicted in 2D crystals, such as MoS_2_, hexagonal boron nitride, and graphene bilayers.^[^ ^49^ ^]^

2D ferroelectric material, as a unique member of 2D material family, may offer new opportunities in superlubricity due to electrically tunable interlayer dipole–dipole coupling which is absent in other types of sliding contacts. Here, we employ first‐principles calculations to predict that negative friction coefficient *μ* can be realized in 2D ferroelectric In_2_Se_3_ commensurate contacts which break the incommensurate contact condition and the classic Da Vinci–Amontons law. The friction force *f*, measured as the potential corrugation or sliding barrier (*E*_bar_)^[^ ^33^, ^34^, ^35^, ^50^ ^]^ as rationalized by the Prandtl–Tomlinson (PT) model, is lowered upon increasing normal load *N*. It is widely accepted that the interlayer potentials or sliding corrugation of 2D junctions are essentially determined by two factors, vdW and electrostatic interactions.^[^ ^51^, ^52^, ^53^ ^]^ Therefore, as long as the delicate interplay of the interfacial vdW force and the electrostatic energy dependent on the dipole–dipole alignments at the ferroelectric 2D contact can be tuned to appropriate regime, negative *μ* is destined to emerge, and the condition of incommensurate contact becomes dispensable. We validated this strategy in the investigation of the sliding friction between two quintuple layers (QL) of 2D ferroelectric In_2_Se_3_^[^ ^54^, ^55^, ^56^, ^57^ ^]^ commensurate contacts with appropriate interlayer dipole–dipole alignments. Here, the vdW interactions enhance the potential energy corrugation (PEC) due to the repulsive regime of the interfacial separation subject to the dipole‐alignment dependent interfacial interactions under load, in contrast to previous findings^[^ ^46^, ^48^ ^]^ that vdW interactions predominantly reduce the PEC in the attractive regime of the interfacial separation above its equilibrium. However, the electrostatic energies reduce the PEC by enhancing the intralayer Se—In ionic bonding due to charge redistribution under load, and the delicate interplay between the vdW interactions and electrostatic energy leads to NDF.

## 2 Results and Discussion

### 2.1 Properties of 1QL‐In_2_Se_3_ and 2QL‐In_2_Se_3_ Systems

**Figure 1:**
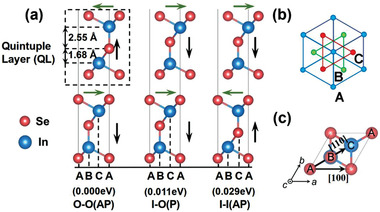
a) The most stable contacts of three typical out‐of‐plane polarization alignments of two quintuple layers (QL) of In_2_Se_3_, i.e., 2QL‐In_2_Se_3_, and b) Top view of one QL along the vertical direction. Each atomic layer in a QL contains only one elemental species, with the atoms arranged in one of the triangular lattices A, B, or C as illustrated. c) Two typical sliding pathways of the top QL In_2_Se_3_ relative to the bottom one, i.e., along [110] and [100], respectively. The relative energies of the three most stable contacts are shown in the brackets, with the most stable O‐O(AP) as the energy reference.

First, the calculated lattice constant of In_2_Se_3_ is 4.11 Å and the optimized thickness of In_2_Se_3_ is about 6.8 Å,^[^ ^54^ ^]^ in close agreement with previous calculations^[^ ^54^ ^]^ and experimental results.^[^ ^55^, ^56^ ^]^ As shown in **Figure** **1**a,b, the significantly different interlayer spacing between the Se layer and the two In layers and the in‐plane centrosymmetry breaking of the 1QL In_2_Se_3_ result in spontaneous out‐of‐plane (≈0.1 eÅ) and in‐plane (≈2.4 eÅ) electric polarizations,^[^ ^54^ ^]^ respectively.

Then, we identify the preferred high‐symmetry stacking modes of the homogeneous contact. As shown in Figure 1, for 2QL In_2_Se_3_ commensurate contacts, in the out‐of‐plane direction, the electric polarization points either inward (I) or outward (O) with respect to the interface. Moreover, for each of such three cases, the in‐plane polarizations of the two QLs can be aligned either in parallel (P) or antiparallel (AP) manners. Therefore, the six configurations of 2QL In_2_Se_3_ can be labeled as O‐O(P), O‐O(AP), I‐O(P), I‐O(AP), II(P), and I‐I(AP), respectively. Accordingly, the most stable high‐symmetry commensurate contacts with three typical out‐of‐plane alignments, i.e., O‐O(AP), I‐O(P), and I‐I(AP) are presented in Figure 1a, and their metastable counterparts, i.e., O‐O(P), I‐O(AP), and I‐I(P) are shown in Figure S1 in the Supporting Information, respectively. As also summarized in the Table S1 (Supporting Information), the binding energies of the most stable three stacking configurations are highly correlated with their interlayer distances (*d*), namely, the stronger the binding of the 2QL contact is, the smaller the *d* becomes, as manifested by the optimized *d* of 2.86, 2.98, and 3.02 Å, for O‐O(AP), I‐O(P), and I‐I(AP), respectively. Such a trend is also observed for the three low‐lying counterparts.

#### 2.1.1 Interlayer Sliding Energy Profile of 2QL‐In_2_Se_3_ Homojunctions Along [110]

Now, starting from the optimized most stable stacking configuration of O‐O(AP), we analyze the energy profile of the interlayer sliding along two typical commensurate pathways, i.e., [110] and [100] directions, where the bottom Se atoms of the top QL (Se_BT_) are displaced straightly from A site, over B and C, to the second‐nearest neighboring A, and directly displaced from an A site to the nearest neighboring A site, as respectively shown in Figure 1c. To mimic the experimental investigations of friction as performed on other 2D materials,^[^ ^35^ ^]^ when sliding the top QL In_2_Se_3_ relative to the bottom QL, the X, Y, and Z coordinates of the bottom‐two layers of atoms in the bottom QL In_2_Se_3_ are fully fixed, and the energy profile of the commensurate contact is optimized by displacing the top QL In_2_Se_3_ every 0.3 Å along the proposed directions with fixing X and Y coordinates of the top‐two layers atoms.^[^ ^34^ ^]^ That is, in the constrained sliding pathways, Z direction of the top‐two layers and X, Y, and Z directions of the six intermediate layers can be fully relaxed under external load.

**Figure 2:**
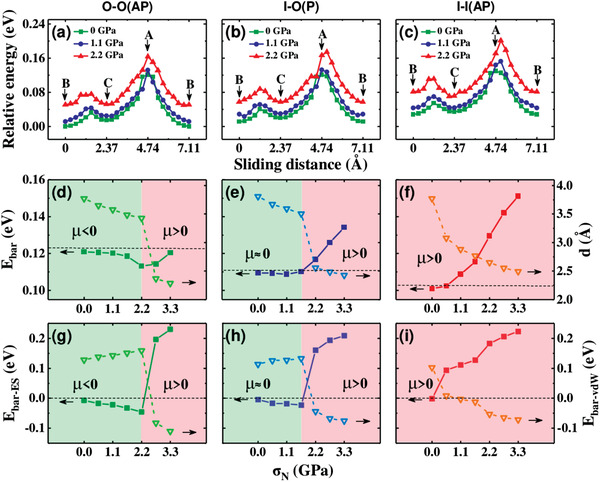
a–c) Load dependent sliding energy profiles, d–f) Sliding energy barriers (*E*_bar_) along [110] pathway as shown in Figure 1c. The contribution of the *E*_bar_ by vdW corrections (*E*_bar−vdW_) and electrostatics interactions (*E*_bar−ES_) are analyzed in g–i). a), d), and g): O‐O(AP); b), e), and h): I‐O(P); c), f), and i): I‐I(AP). In d–f), the interface separation distance d of the *E*_max_ configuration of the three contacts are also presented.

The calculated energy profiles of the three stable commensurate contacts of O‐O(AP), I‐O(P), and I‐I(AP) along the [110] pathway under three representative loads, i.e., *N* = 0.00, 1.1, and 2.2 GPa, are presented in **Figure** **2**a–c, respectively. Two distinct features can be observed: i) In all the three contacts, during the whole sliding period, two local maxima of energy are identified, with the highest energy maximum (*E*_max_) occurred when Se_BT_ is slid to around the A site; ii) With the external *N* increasing, the *E*_max_ can be slightly pass over the A site, and the lowest energy minimum (*E*_min_) may be shifted from B site to C, as found in the case of I‐I(AP) with *N* = 2.2 GPa. Correspondingly, we present the activation energy barrier (*E*_bar_) of the interlayer sliding subject to different loads in Figure 2d–f, for O‐O(AP), I‐O(P), and I‐I(AP), respectively. Here, we define the *E*_bar_ as the energy difference between the *E*_max_ and *E*_min_. Dividing the *E*_bar_ by the sliding distance,^[^ ^64^ ^]^ we can estimate the friction force *f* during the sliding. Very interestingly, the three types of commensurate contacts possess very contrasting *E*_bar_ (*f*) curves as a function of load *N*, as shown in Figure 2d–. Specifically, for O‐O(AP), the *E*_bar_ (*f*) decreases with *N* increasing, namely, the differential friction coefficient *μ* = ∆*f*/∆*N* <0 can be identified in a fairly large load regime up to 2.2 GPa applicable in recent experiments on 2D material contacts.^[^ ^12^, ^45^ ^]^ Here, we emphasize that the NDF in the present load regime is also confirmed by further calculations performed by using HSE+D3 and optPBE‐vdW schemes, as detailed in Figure S2 in the Supporting Information. However, *μ* > 0 is observed when *N* is beyond 2.2 GPa. Note that, here the predicted overall variation of friction with normal load is estimated to be about several nN according to the definition;^[^ ^34^ ^]^ importantly, the variation of the friction within 0–2.2 GPa reaches up to about 6.4%. Therefore, we expect that the NDF in the present work can be probed by the elaborate experiments.^[^ ^38^ ^]^

**Figure 3:**
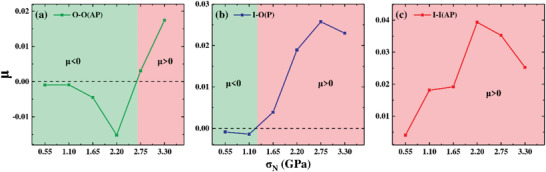
Calculated friction coefficient *µ* (*µ* = ∆*f* /∆*N*). a) O‐O(AP); b) I‐O(P); c) I‐I(AP).

Moreover, we have also calculated the differential friction coefficients *μ* (*μ* = ∆*f* /∆*N*) for all the three representative systems. As shown in **Figure** **3**a–c, it is very clear that for the case of O‐O(AP), the friction coefficient *μ* is indeed negative in the load regime of 0 < *N *≤ 2.2 GPa, with a maximum absolute value of 0.015 at 2.2 GPa; for the case of I‐O(P), *μ* is negative (with relatively small absolute values) in the load regime of *N* ≤1.10 GPa; In contrast, for I‐I(AP), *μ* is positive and increases up to the maximum absolute value of about 0.04 at 2.2 GPa, beyond which the calculated *μ* begins to decrease with the load, corresponding to the *α*‐to‐*β*‐like structural distortion. These findings provide strong evidence that the electric dipole can serve as an important degree of freedom to modulate the differential friction coefficient of In_2_Se_3_ interlayer commensurate sliding, i.e., from *μ *< 0, through *μ *≈ 0, to *μ *> 0.

#### 2.1.2 Origin of the Negative Differential Friction Coefficient µ

To rationalize the above intriguing findings, we decompose the calculated *E*_bar_ (*f*) into two terms which are contributed purely by vdW interactions (*E*_bar−vdW_) and electrostatics interactions (*E*_bar−ES_),^[^ ^51^, ^52^, ^53^ ^]^ as shown in Figure 2g–i for O‐O(AP), I‐O(P), and I‐I(AP), respectively. Here, the *E*_bar−vdW_ is defined as the energy difference of the vdW correction energies between *E*_max_ and *E*_min_, and attribute the left portion of the *E*_bar_ to electrostatics interactions, respectively. As seen from Figure 2g,h, for O‐O(AP)(I‐O(P)) commensurate contact, the *E*_bar−vdW_ gradually increases with load *N* increasing up to about 2.2 (1.65) GPa, beyond which the *E*_bar−vdW_ begins decreasing. Nevertheless, the *E*_bar−ES_ exhibits totally reversed trend in the same load regime, i.e., decreases within ≈0–2.2 (1.65) GPa, and increases beyond 2.2 (1.65) GPa, respectively. Clearly, the negative (quasizero) *μ* of O‐O(AP) (I‐O(P)) can be ascribed to the more significant (quasi‐identical) reduction of the *E*_bar−ES_ which compensates the raise of the *E*_bar−vdW_. More detailed analysis in Figure S3 in the Supporting Information show that the Ewald energy and exchange correlation potential dependent on the charge transfers and charge redistributions under load dominate the *E*_bar_ reduction and negative friction. For I‐I(AP), in the whole load regime of ≈0–3.3 GPa, the *E*_bar−vdW_ exhibits monotonous decrease which nevertheless is insufficient to compensate the monotonous increase of the *E*_bar−ES_, leading to a reversed sign of the *μ*, i.e., *μ *> 0.

#### 2.1.3 Role of Interlayer vdW Interactions Verses Intralayer Electrostatic Interactions

Here we emphasize that though NDF arises from the decreased sliding potential corrugation with increased normal load is also observed in other systems,^[^ ^45^, ^46^, ^47^, ^48^ ^]^ however, such NDF is dominated by van der Waals interactions in the attractive regime of the interfacial separation above its equilibrium.^[^ ^46^, ^48^ ^]^ However, in our findings, the vdW interactions increase, rather than decrease the potential corrugation with the load increasing, very probably due to the fact that the interlayer distances are reduced to the sharp repulsive regime of the interfacial vdW interactions, as supported by the smallest interfacial separations in the OO(AP) and detailed analysis in Figure S4 in the Supporting Information. These findings demonstrate the critical role of the electrostatic interactions in dominating the NDF of the ferroelectric 2D material In_2_Se_3_ vdW commensurate contact. Moreover, as detailed in Figure S5 in the Supporting Information, the increase of the friction under load beyond 2.75 GPa in O‐O(AP) is accompanied with a structure distortion of the top QL In_2_Se_3_ in the *E*_max_ state, and an *α* to *β* phase transition is observed beyond 3.3 GPa. Such a transition results in energy increases, due to the higher energy of the *β* phase than *α*, by about 0.01 eV for the 1QL In_2_Se_3_^54^ under the present pressure regime, as also detailed in Figure S6 in the Supporting Information. For I‐O(P), such a structural distortion and phase transition begins from relatively small critical load, around 1.65 GPa. However, no significant phase transition is observed in both the *E*_max_ state of I‐I(AP) contact and *E*_min_ states of all the three systems.

**Figure 4:**
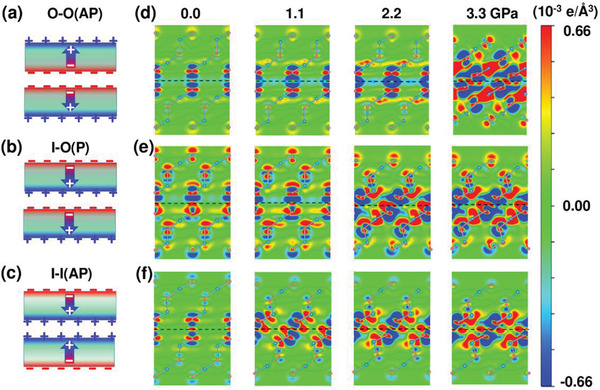
a–c) Schematic shows of the interactions of the interfacial polarization charges of the *E*_max_ configurations. d–f) 2D charge difference counters in the (110) plane consisting of the [110] pathway are presented for all the three systems under *N* = 0, 1.1, 2.2, and 3.3 GPa. The black dashed lines represent the middle of the interfaces. a) and d): O‐O(AP); b) and e): I‐O(P); c) and f): I‐I(AP).

To illustrate the underlying mechanism of the specific alignment of ferroelectric polarizations in leading to distinct frictions of the present systems, we first analyze the energy band structure of 1QL In_2_Se_3_ in Figure S7 in the Supporting Information, which exhibits semiconducting characteristic with an energy bandgap of 0.78 eV. Due to the out‐of‐plane polarization, there are positive and negative charges localized respectively on the top and bottom sides of a free standing 1QL In_2_Se_3_ as defined in Figure 1a. Such a feature is also supported by the centrosymmetry breaking nature (along the *z* direction) of the electronic charge density of the valance band, which is mainly localized on the top surface, i.e., the starting side of the electric dipole, see Figure S8 in the Supporting Information. Correspondingly, the interfacial couplings of the three 2QL In_2_Se_3_ commensurate contacts can be schematically simplified as models of two contacting surfaces carrying positive and/or negative polarization charges, as shown in the left panels in **Figure** **4**a–c, for O‐O(AP), I‐O(P), and I‐I(AP), respectively.

The contrast friction behaviors of the three systems can be readily understood from the distinct responses of these polarization charges at the *E*_max_ configurations under load. Specifically, for O‐O(AP), due to the electrostatic repulsive interactions of the negative charge themselves and the experienced electric field force applied by the electric dipole of the opposite QL, part of the surface polarization negative charges are respectively repelled to the nearby Se atom sites, as demonstrated by the charge difference analysis in Figure 4d. Such a charge accumulation (in red) on Se atoms lowers the electrostatics energies of the two individual QL components and thus reduces the *E*_bar−ES_ via enhancing the Se—In ionic binding. Importantly, such a charge transfer is significantly enhanced subject to the load *N* increase. For examples, the *E*_bar−vdW_ increases from 0.129 to 0.159 eV, however the *E*_bar−ES_ decrease from −0.008 to −0.046 eV when *N* increases from 0 to 2.2 GPa, and it is clear that the *E*_bar−ES_ decrease overtakes the *E*_bar−vdW_ increase by 0.006 eV. For I‐O(P) shown in Figure 4e, in the load regime of ≈0–1.1 GPa, the enhancement of In—Se ionic bonding induced by the charge transfer can only be observed in the bottom QL, however it is weakened in the top QL, which leads to quasizero friction coefficient. For I‐I(AP) shown in Figure 4f, on the one hand, with the interlayer distance decreasing under load, the repulsive electrostatic interactions of the positive polarization charges on both sides of the interfaces increases, leading to the distortion of the interface. On the other hand, more electrons are attracted to the central regime of the interface from the two individual QL components under load which lowers the electrostatic potential barrier of the interface as illustrated in Figure S9 in the Supporting Information. In contrast to O‐O(AP), such a reversed charge transfer in I‐I(AP) from both the top and bottom QLs to the interface regime significantly lowers the intralayer binding of each QL component at *E*_max_. Collectively, such two factors result in enlarged potential corrugation and positive *μ* in I‐I(AP) contact.

### 2.2 Interlayer Sliding Energy Profile of 2QL‐In_2_Se_3_ Homojunctions Along [100]

Now, we briefly report that when the top QL In_2_Se_3_ is displaced under external load along the [100] direction, though the X and Y coordinates of the top‐two layers of atoms are fixed along the [100] direction, the six intermediate layers of atoms will be optimized to close the configuration as obtained that along the [110] pathway. Such a departure from the [100] to the [110] pathway of the intermediate atoms leads to instability of the contact, particularly at relatively large load regime shown in Figure S10 in the Supporting Information. These findings suggest that the interlayer sliding prefers to proceed along the present “guide rail,” i.e., [110] direction, probably due to the significantly anisotropic features of the potential surface, which facilitates in realizing superlubricity.^[^ ^45^ ^]^

### 2.3 Role of External Electric Field in Tuning the Friction

**Figure 5:**
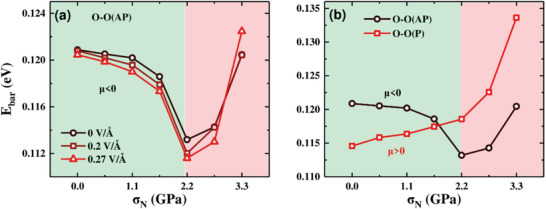
Sliding *E*_bar_ of a) O‐O(AP) in electric fields of 0, 0.20, and 0.27 V Å^−1^, b) O‐O(AP) versus O‐O(P) subject to load *N*.

To the end, taking the most stable contact O‐O(AP) as a typical example, we briefly highlight that the new strategy established here in modulation of the friction by ferroelectric polarization can be further convincingly demonstrated by applying external electric field which can modify the out‐of‐plane polarization and/or by changing the in‐plane polarization arrangement of the two QLs as well. As shown in **Figure** **5**a, for the case of O‐O(AP), under positive electric fields, along the [110] direction, the *E*_bar_(*f*) can be further lowered and the negative friction coefficient feature becomes more significant in the relatively low load regime (*N *< 2.2 GPa), which can be also rationalized by the interfacial polarization charges changes under the external electric field, as detailed in Figure S11 (Supporting Information). Moreover, as shown in Figure 5b, the sliding *E*_bar_(*f*) in O‐O(AP) can be significantly lowered via reversing the in‐plane dipole alignment to parallel arrangement, i.e., O‐O(P), however leading to positive *μ* in the whole present load regime. These findings suggest that In_2_Se_3_ with controllable friction is highly desirable in design of functional nanodevices wherein tunable friction is demanded.

## 3 Conclusion

In conclusion, the present study predicts that negative sliding friction coefficient can be realized in electrically polarized 2D material *commensurate* contacts. By selecting appropriate dipole–dipole alignments between the individual components of the contact, the raise of the sliding energy barrier contributed by vdW interactions can be thoroughly compensated by the electrostatic energy reduction, which is a spontaneous polarization charge redistribution process under appropriate load regime. As a consequence, negative friction coefficient can be obtained in the commensurate contact, and incommensurate condition is broken. Also importantly, the new strategy established here in reversion of the sliding friction coefficient by electric‐dipole is also demonstrated by applying out‐of‐plane electric field and/or by changing the in‐plane ferroelectric polarization alignments. Due to the fundamental importance, the present central findings are also expected to play an instrumental role in developing functional lubrications, nanosensors, and related mechanical‐electronic nanodevices based on ferroelectric materials.

## 4 Experimental Section

The calculations were carried out using the density functional theory (DFT)^[^ ^58^ ^]^ as implemented in the VASP code.^[^ ^59^ ^]^ The interaction of valence electrons with atomic cores is described by the projector‐augmented wave (PAW) method,^[^ ^60^ ^]^ as parameterized by the Perdew‐Burke‐Ernzerhof (PBE) functional.^[^ ^61^ ^]^ It is demonstrated that, relative to other vdW correction schemes (such as many‐body dispersion which is powerful in describing other systems^[^ ^34^, ^62^ ^]^), semiempirical DFT‐D3 method^[^ ^63^ ^]^ can more accurately describe the *α* configuration of the ground sate of 1QL‐In_2_Se_3_.^[^ ^54^ ^]^ More calculation parameters and testing computational details are provided in Figures S1 and S6 in the Supporting Information. For 2D systems, the out‐of‐plane electric polarization is well‐defined following the classical electrodynamics and calculated by the integration of local charge density times the coordinate in the out‐of‐plane axis over the whole supercell.^[^ ^54^ ^]^

## Conflict of Interest

The authors declare no conflict of interest.

## Supporting information

Supporting Information

## Data Availability

Research data are not shared.
