# Supplementary material for: Negative Differential Friction Predicted in 2D Ferroelectric In2Se3 Commensurate Contacts
Source: Adv Sci (Weinh). 2021 Nov 10;9(2):2103443. doi: 10.1002/advs.202103443 (PMC8805561; doi:10.1002/advs.202103443)
Supplement: Supplementary file 1 — Supporting Information [file ADVS-9-2103443-s001.pdf]

© 2021 Wiley-VCH GmbH

ADVANCED SCIENCE
Open Access

Supporting Information

for *Adv. Sci.*, DOI: 10.1002/advs.202103443

Negative Differential Friction Predicted in Two-dimensional Ferroelectric In_2Se_3 Commensurate Contacts

Jingge Sun, Lili Zhang, Rui Pang, Xing-Ju Zhao, Jiangtao Cheng, Yimin Zhang, Xinlian Xue, Xiaoyan Ren, Wenguang Zhu,* Shunfang Li,* and Zhenyu Zhang*

Supplementary Materials for

Negative Differential Friction Predicted in Two-dimensional Ferroelectric In_2Se_3 Commensurate Contacts

Jingge Sun,[1] *Lili Zhang,*[1] *Rui Pang,*[1] *Xing-Ju Zhao,*[1] *Jiangtao Cheng,*[1] *Yimin Zhang,*[1] *Xinlian Xue,*[1] *Xiaoyan Ren,*[*,1] *Wenguang Zhu,*[*2,3] *Shunfang Li,*[*,1] *and Zhenyu Zhang*[3]

[1]Key Laboratory of Material Physics, Ministry of Education, School of Physics and Microelectronics, Zhengzhou University, Zhengzhou 450001, China.

[2]Key Laboratory of Strongly-Coupled Quantum Matter Physics, Chinese Academy of Sciences, School of Physical Sciences, University of Science and Technology of China,

Hefei, Anhui 230026, China

[3]International Center for Quantum Design of Functional Materials (ICQD), Hefei National Laboratory for Physical Sciences at the Microscale, and Synergetic Innovation Center of Quantum Information and Quantum Physics, University of Science and Technology of China, Hefei, Anhui 230026, China

E-mail: renxyan@zzu.edu.cn; wgzhu@ustc.edu.cn; sflizzu@zzu.edu.cn

This file includes:

Fig. S1. Configurations of the metastable contacts, O-O(AP), I-O(P), and I-I(AP), with the relative energies are presented in the brackets.

Table S1. Binding energies (E_b) and interlayer distance (d) of the six typical contacts.

Fig. S2. Calculated total E_{bar} and decomposed $E_{bar\text{-}vdW}$ and $E_{bar\text{-}ES}$ for the O-O(AP) 2QL-In_2Se_3 interlayer sliding. **(a)** and **(c)** calculated by HSE+D3; **(b)** and **(d)** by optPBE-vdw.

Fig. S3. Load dependent E_{bar} decomposed from **(a)** Hartree energy, **(b)** ion-ion interactions, i.e., Ewald energy, **(c)** Coulomb interactions of electron-electron and electron-ion, and **(d)** Exchange correlation energy and exchange correlation potential,

i.e., -V(xc)+E(xc). The calculations are performed with PBE+D3 method, which are the same as that presented in **Fig. 2(g)**.

Fig. S4. (a) Charge states of each In and Se atoms in the 1QL-In_2Se_3. **(b)** Optimized interlayer distances of 2QL- In_2Se_3 in the stackig forms of O-O(AP), I-O(P) and I-I(AP). **(c)** Schematic illustration the role of vdW interactions in changing the potentail corrugations of the 2QL-In_2Se_3 under external load, which reduces the interfacial separation distance *d*.

Fig. S5. Schematic show of the phase transition In_2Se_3 at the E_{max} state under load.

Fig. S6. Comparison of the total energies of 1QL-In_2Se_3 between α and β phases calculated with (a) PBE+D3, (b) HSE+D3, (c) optPBE-vdW; (d) PBE+MBD, (e) HSE+MBD.

Fig. S7. Energy band structure of 1QL α-In_2Se_3.

Fig. S8. Projected three-dimensional electronic charge density of the valance band of 1QL α-$In_2Se_{3,}$ with the isosurface value of 0.003 e/$Å^3$.

Fig. S9. Electrostatic potentials of the three most stable contacts.

Fig. S10. Energy profiles and geometric structures of the O-O(AP) contact when sliding the top QL In_2Se_3 relative to the bottom QL along [110] and [100] without load.

Fig. S11. Interfacial polarization charge changes of the E_{max} state of O-O (AP) under the external electric fields (EEF) and external loads. **(a)** Without electric field; **(b)** With electric field, EEF=0.27 V/Å.

S1. More calculation details.

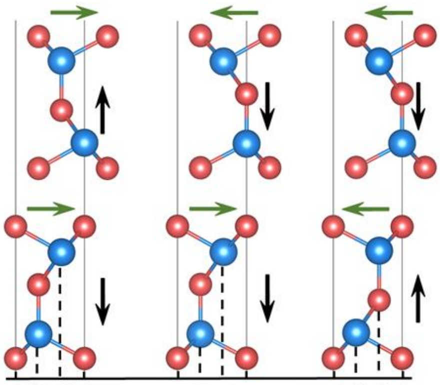

FIG. S1. Configurations of the metastable contacts, O-O(P), I-O(AP), and I-I(P), with the relative energies are presented in the brackets.

In the **Fig. S1**, the configurations of the three metastable contacts, O-O(P), I-O(AP), and I-I(P) are presented with the relative energies shown in the brackets. Specifically, the optimized O-O(P), I-O(AP), and I-I(P) stacking forms are 0.008, 0.015 and 0.031 eV higher in energy relative to the ground state structure of O-O(AP) shown in **Figure 1(a)**.

Table S1. Binding energies (E_b) and interlayer distance (d) of the six typical contacts.

**Contacts**	**E_b (eV)**	**d (Å)**
O-O(AP)	-1.758	2.86
O-O(P)	-1.750	2.99
I-O(P)	-1.747	2.98
I-O(AP)	-1.742	3.01
I-I(AP)	-1.729	3.02
I-I(P)	-1.727	3.05

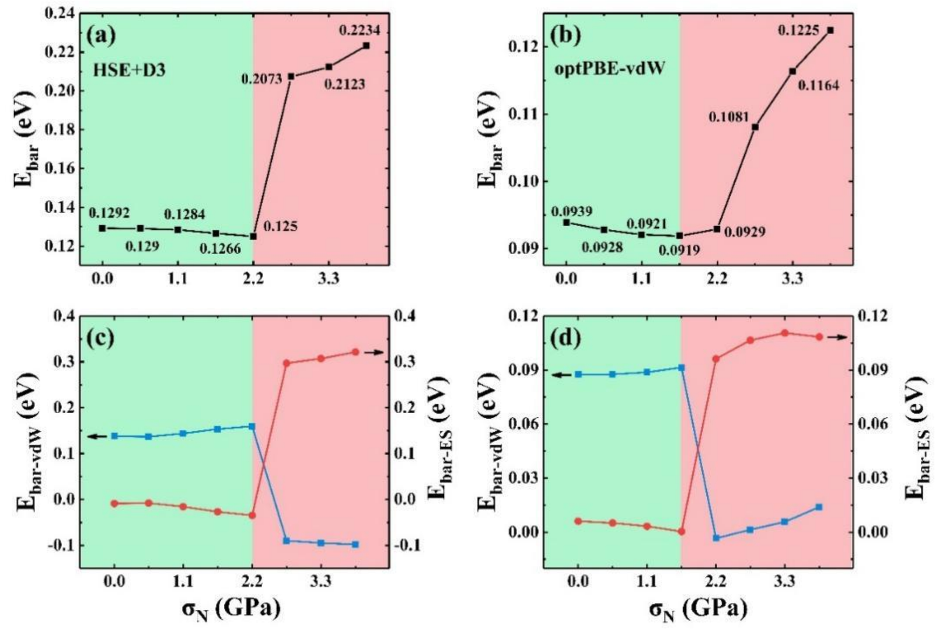

FIG. S2. *Calculated total E_{bar} and decomposed $E_{bar\text{-}vdW}$ and $E_{bar\text{-}ES}$ for the O-O(AP) 2QL-In_2Se_3 interlayer sliding.* ***(a)*** *and* ***(c)*** *calculated by HSE+D3;* ***(b)*** *and* ***(d)*** *by optPBE-vdw.*

As shown in Fig. S2, we have further examined our central findings by using both **HSE+D3 and optPBE-vdW** methods, and qualitatively confirmed the quite universal negative friction behavior of the present 2D ferroelectric material. As seen from **FIG. S2 (a) and 2(b)**, both HSE+D3 and optPBE-vdW methods can also qualitatively reproduce negative friction properties of the O-O(AP) commensurate contact. Specifically, in the load regime of 0～2.2GPa, the sliding barrier (E_{bar}) is reduced respect to the load increasing, leading to negative μ, though the slope of the curves are relatively reduced as compared to the case of PBE+D3 method. Note that, for HSE+D3 and optPBE-vdW calculations, the lattice constants of the 1QL-In_2Se_3 are re-optimized and used for the 2QL-In_2Se_3 homojunctions to investigate the friction properties.

Also importantly, as shown in **FIG. S2(c)** and **2(d)**, detailed analysis further confirmed that the vdW interactions of the 2QL-In_2Se_3 contact increase, rather than reduce the E_{bar}. Particularly, the net reduction of the E_{bar} is also originated from the delicate interplay of the vdW interaction and electrostatic energy of the polarized 2D In_2Se_3 interlayers, namely, the latter reduces the E_{bar} and dominates the negative friction behavior of the present ferroelectric 2D material.

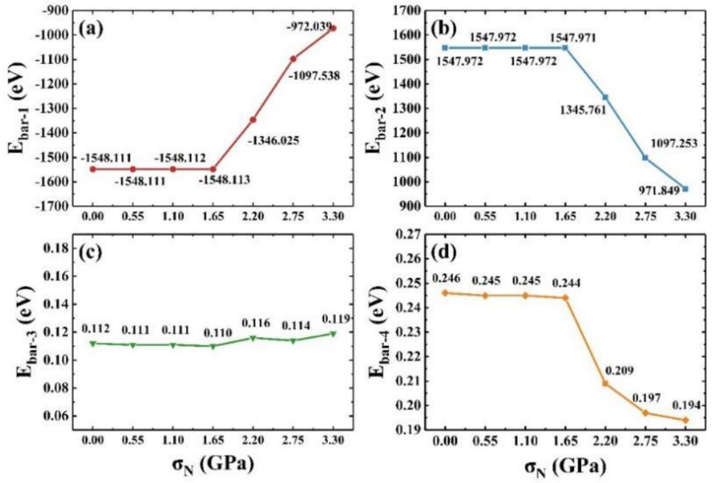

FIG. S3. *Load dependent E_{bar} decomposed from (a) Hartree energy, (b) ion-ion interactions, i.e., Ewald energy, (c) Coulomb interactions of electron-electron and electron-ion, and (d) Exchange correlation energy and exchange correlation potential, i.e., -V(xc)+E(xc). The calculations are performed with PBE+D3 method, which are the same as that presented in* ***Fig. 2(g)***.

Here, we have performed more detailed analysis on the origin of the E_{bar}, as shown in **FIG. S3**. One can see that in the load regime of 0～1.65 GPa, both Hartree energy and Coulomb interactions of electron-electron and electron-ion lead to negligible contribution to E_{bar} reduction. On the contrary, both terms raise the E_{bar} when the load increases from 1.65 to 2.20 GPa, by about 202.088 and 0.006 eV, respectively. However, in the same load regime of 1.65-2.20 GPa, another two terms, including both Ewald energy and exchange correlation energy (-V(xc)+E(xc)), reduces the E_{bar} by 202.211 and 0.035 eV, respectively, unambiguously, these two terms together dominate the negative friction.

Here, we group these two terms (Ewald energy and the term of -V(xc)+E(xc)) into the framework of electrostatic interactions which are charge transfer and redistribution dependent under external load.

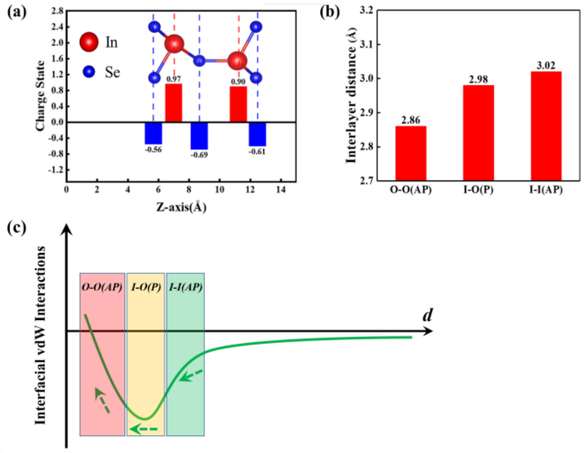

FIG. S4. ***(a)*** *Charge states of each In and Se atoms in the 1QL-In_2Se_3.* ***(b)*** *Optimized interlayer distances of 2QL- In_2Se_3 in the stackig forms of O-O(AP), I-O(P) and*

I-I(AP). ***(c)*** *Schematic illustration the role of vdW interactions in changing the potentail corrugations of the 2QL-In_2Se_3 under external load, which reduces the interfacial separation distance d.*

Here, we demonstrate the above argument that, in O-O(AP) with NFC, **the interfacial vdW interactions are in the repulsive regime.** As shown in **FIG. S4(a)**, in the 1QL ferroelectric In_2Se_3, along the normal direction of the 1QL-In_2Se_3, from left to right the five atoms is charged by 0.56, 0.97, 0.69, 0.90 and 0.61 electrons on the In cations and Se anions, respectively. Therefore, when 2QLs of In_2Se_3 are staked in the form of O-O(AP), I-O(P), and I-I(AP), as shown in the **Figure 1** of the main text, the local electrostatic repulsive interaction strength of the interfacial Se anions of the 2QL-In_2Se_3 of the three staking forms are in the order of O-O(AP)<I-O(P)<I-I(AP), which leads to the same order sequence for the interfacial separation distance *d* of the three cases, as shown in **FIG. S4(b)**. Specifically, O-O(AP) and I-I(AP) possesses the smallest and the largest interfacial separations, 2.86 and 3.02 Å, respectively. Therefore, as schematically shown in **FIG. S4(c)**, under external load, the further reduced interfacial separation distance *d* of the O-O(AP) will readily lies in the ***sharp repulsive regime*** of the vdW interactions, which enhances the potential corrugation; nevertheless, for the case of I-I(AP), under external load up to 2.2 GPa, the reduced interfacial separation *d* may still lies in the relatively smooth attractive regime of the vdW interactions, leading to reduced potential corrugation for the vdW interactions, as demonstrated by **Figures 2(g) and 2(i)**, respectively.

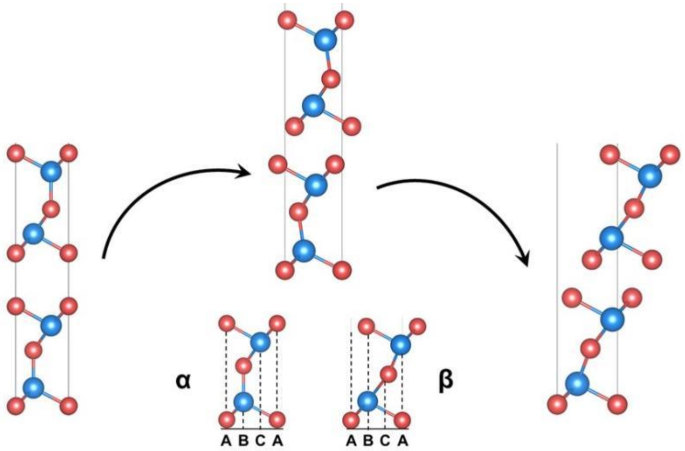

FIG. S5. Schematic show of the phase transition In_2Se_3 at the E_{max} state under load.

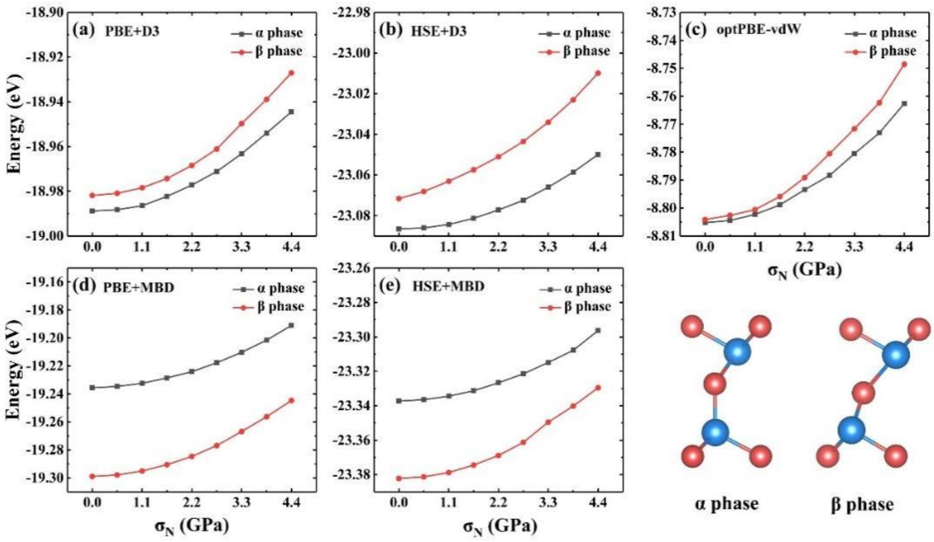

FIG. S6. *Comparison of the total energies of 1QL-In_2Se_3 between α and β phases calculated with **(a)** PBE+D3, **(b)** HSE+D3, **(c)** optPBE-vdW; **(d)** PBE+MBD, **(e)** HSE+MBD.*

We have also examined our findings by using other functionals, such as the HSE+MBD approach. Unfortunately, we confirm that the MBD scheme is invalid in correctly describing the ground state feature of the 1QL-In_2Se_3 system.

In doing this, we first examined the relative stability of the 1QL- In_2Se_3 system in α and β phases. As shown in **Figure S6(a)**, one can see that the PBE+D3 approach can correctly demonstrate the α phase feature of the ground state 1QL- In_2Se_3, as clearly manifested by the uniformly lower total energy of the α phase as compared to the β phase in all the load regime as investigated in the present work, which is also supported by the calculations performed by using HSE+D3 (see **Figure S6(b)**) and optPBE-vdW (**Figure S6(c)**) methods. Note that, the α phase has been well established to be the ground state structure of In_2Se_3, as supported by previous theoretical calculation [Ding et al., Nat. Commun. 8, 14956 (2017)] and experimental observations [Zhou et al., Nano Lett. 17, 5508 (2017); Cui et al., Nano Lett. 18, 1253 (2018); Xiao et al., Phys. Rev. Lett. 120, 227601 (2018)].

However, as shown in **Figure S6 (d) and 6(e)**, in both PBE+MBD and HSE+MBD calculations, the relative stability of the 1QL-In_2Se_3 is reversed between α and β phases, namely, β phase is now lower in energy than α phase. Therefore, we can conclude that MBD method is not suitable to describe the ground state structure of 1QL-In_2Se_3, and thus we don't think it can correctly describe the interlayer interactions and the friction property of the 2QL-In_2Se_3 homojunctions.

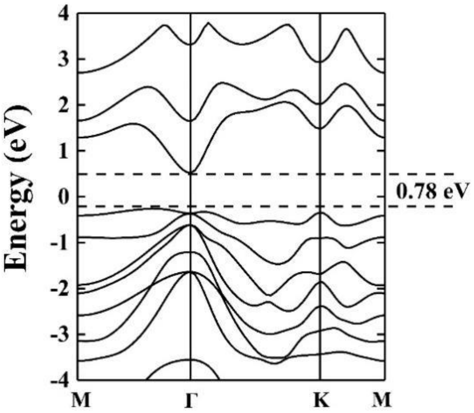

FIG. S7. Energy band structure of 1QL α-In_2Se_3.

From the electronic band structure, one can see that the band gap of the 1QL α-In_2Se_3 is 0.78 eV calculated by PBE functional.

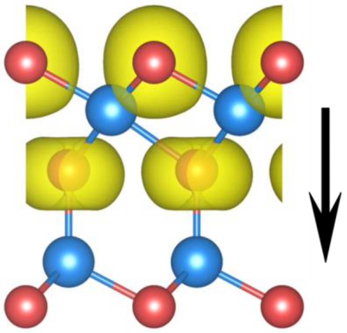

FIG. S8. Projected three-dimensional electronic charge density of the valance band of 1QL α-In_2Se_3, with the isosurface value of 0.003 e/Å^3.

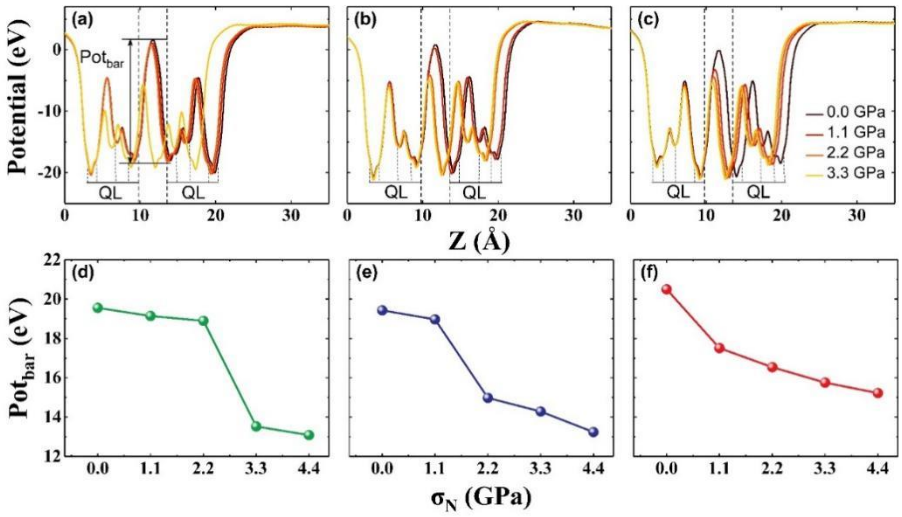

FIG. S9. Electrostatic potentials and Pot_{bar} of the three most stable contacts under external loads **(a)** and **(d)** O-O(AP), **(b)** and (e) I-O(P), **(c)** and **(f)** I-I(AP). Pot_{bar} is

defined as the potential difference between the minimum and maximum of the potential in the vicinity of the interface.

From **Fig. S9 (d)** and 9**(f)**, one can see that, as compared to the case of O-O(AP), the Pot_{bar} in I-I(AP) is reduced more significantly under loads within the regime of 0~2.2 GPa. Such a feature facilitates more charge transfer from each QL structure to the interface regime in the O-O(AP), which significantly lowers the intralayer binding of each QL component and results in higher energy in the E_{max}, leading to positive friction behavior.

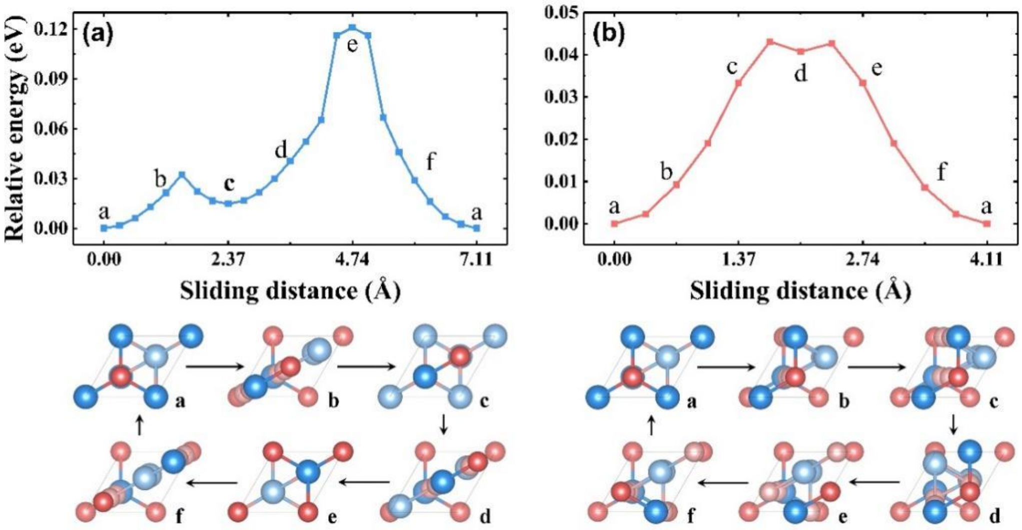

FIG. S10. Energy profiles and geometric structures of the O-O(AP) contact when sliding the top QL In_2Se_3 relative to the bottom QL along **(a)** [110] and **(b)** [100] without load.

In **Fig. S10**, we present the energy profiles and geometric structures of the O-O(AP) contact when sliding the top QL In_2Se_3 relative to the bottom QL along [110] and [100] without load. Though the X and Y coordinates of the top-two layers of atoms are fixed along the [100] direction, the six intermediate layers of atoms will be optimized to close the configuration as obtained that along the [110] pathway. Such a departure from the [100] to the [110] pathway of the intermediate atoms leads to instability of the contact, particularly at relatively large load regime.

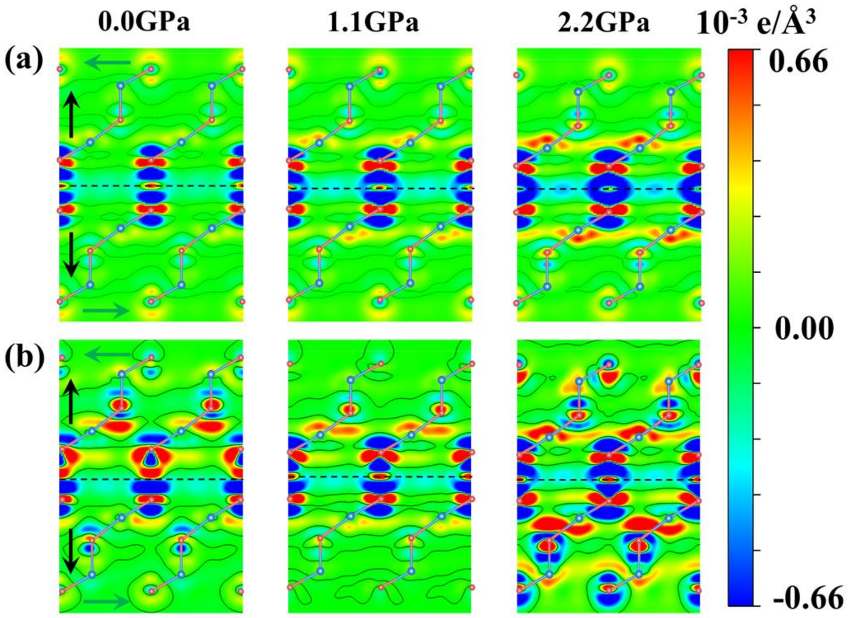

Fig. S11. Interfacial polarization charge changes of the Emax state of O-O (AP) under the external electric fields (EEF) and external loads. **(a)** Without electric field; **(b)** With electric field, EEF=0.27 V/Å.

Here, two-dimensional charge difference counters in the (110) plane consisting of the [110] pathway are presented for O-O (AP) contact under N =0, 1.1, and 2.2 GPa. The black dashed lines represent the middle of the interfaces. As shown in **Figure S11**, we comparatively present the charge difference of the E_{max} states of the O-O(AP) 2QL-In_2Se_3 contact with and without electric field under external load. One can see that, for O-O(AP) without load and external electric filed (EEF), due to the electrostatic repulsive interactions of the negative charge themselves and the experienced electric field force applied by the electric dipole of the opposite QL, part of the surface polarization negative charges are respectively repelled to the nearby Se atom sites, as demonstrated by the charge difference analysis in **Figure S11** (see also **Figure 4d**). Upon the introduction of external electric filed, such as EEF=0.27 V/ Å, the polarization charge localized on the bottom QL-In_2Se_3 exhibits slight changes (see the left panels in **Figure S11(a)** and **(b)**), however, the charge within the top QL-In_2Se_3 changes considerably. Specifically, more negative electron charge is significantly accumulated into the interfacial regime migrated from the bottom layer of In atoms in the top QL- In_2Se_3, meanwhile, some surface polarization charge around the top layer of Se atoms in the bottom QL-In_2Se_3 is shifted into the bottom substrate. Collectively, such a charge redistribution on the one hand reduces the interfacial Coulomb interactions, on the other hand, the charge accumulations between the Se and In atoms enhances the In-Se covalent bonding, which effectively lowers the totally energy of the E_{max} configuration and thus reduces the sliding barrier and friction, as seen from **Figure 5(a)** in the main text.

Moreover, when applied external load, such as 1.1 and 2.2 GPa, the interfacial polarization charge can be further squeezed into the two QLs, considerably enhancing the In-Se covalent (somewhat ionic) bonding, further lowering the total energy of the E_{max} states and leading to more significant NDF (see **Figure 5(a)**), in consistent with the physical picture delivered in the main text of **Page 9-Page 10.**

S1. More calculation details.

In our calculations, the electronic wave functions are expanded in a plane-wave basis with an energy cutoff of 250 eV. To model the friction of one In_2Se_3 QL over another QL, the supercells contain a unit cell of two QLs structure with a vacuum region of more than 15 Å. A saw-like self-consistent dipole layer was placed in the middle of the vacuum region to adjust the misalignment between the vacuum levels on the different sides of the film due to the intrinsic electric polarization. A Γ-centered 12×12×1 Monkhorst-Pack k-mesh was used for k-point sampling to converge the total energy within 10^{-5} eV.
